# Translational Considerations in the Development of Intranasal Treatments for Epilepsy

**DOI:** 10.3390/pharmaceutics15010233

**Published:** 2023-01-10

**Authors:** Richard N. Prentice, Shakila B. Rizwan

**Affiliations:** School of Pharmacy, University of Otago, Dunedin 9016, New Zealand

**Keywords:** epilepsy, seizure, intranasal, anti-seizure medication, nose-to-brain, olfactory, drug delivery

## Abstract

Epilepsy is a common and serious neurological disorder, to which a high proportion of patients continue to be considered “drug-resistant”, despite the availability of a host of anti-seizure drugs. Investigation into new treatment strategies is therefore of great importance. One such strategy is the use of the nose to deliver drugs directly to the brain with the help of pharmaceutical formulation to overcome the physical challenges presented by this route. The following review explores intranasal delivery of anti-seizure drugs, covering the link between the nose and seizures, pathways from the nose to the brain, current formulations in clinical use, animal seizure models and their proposed application in studying intranasal treatments, and a critical discussion of relevant pre-clinical studies in the literature.

## 1. Introduction

Epilepsy is defined as a disorder of the brain, characterised by an enduring predisposition to generate epileptic seizures and by the neurobiologic, cognitive, psychological, and social consequences of this condition. The condition therefore requires the occurrence of more than one epileptic seizure, an event that is defined as a transient occurrence of signs and/or symptoms due to abnormal, excessive, or synchronous neuronal activity in the brain [1]. A seizure may be generalised, as is the most recognised presentation involving convulsions, but in a lot of cases may be, at least initially, focal in nature [2,3].

Epilepsy is a disease of all ages, affecting up to 70 million people worldwide [4,5,6], and it comes with the huge burdens of reduced quality of life, high unemployment rates, reduced life expectancy, and comorbidities such as depression [7,8]. Despite decades of international research towards developing pharmacological treatments and the current availability of over 22 anti-seizure medications (ASMs) [9], it is disconcerting to reflect on the statistic that approximately 30% of patients still fall under the classification of “drug-resistant” [4,10], with temporal lobe epilepsy thought to be the most susceptible [11].

Drug resistance is defined by the International League Against Epilepsy (ILAE) as the failure of adequate trials of two tolerated and appropriately chosen and used ASM schedules, whether as monotherapies or in combination to achieve sustained seizure freedom [12]. Proposed mechanisms of drug resistance have been discussed in detail elsewhere [4,10,13,14] but, in general, may involve genetic variation, disease-related mechanisms (seizure aetiology, progression of disease, neural network changes, alterations in drug target(s), alterations in drug uptake into the brain), or drug-related mechanisms (tolerance or ineffective mechanism of action) [4].

Several strategies have been suggested to develop better treatments to address drug resistance. The most widely recognised is the need to develop and utilise broader, goal-oriented models in screening protocols [13]. This is because most ASMs on the market were initially selected for development based on successful performance in the Maximal Electroshock Seizure (MES) and/or the subcutaneous (s.c.) pentylenetetrazole (PTZ) tests. This sent ASM discovery down a multi-decade road of unearthing similar compounds to those that were already used and disregarding compounds that may have been effective through unique mechanisms and might have been of benefit to the large “drug-resistant” population [15,16]. As well as developing screening models relevant to drug resistance, there is also a focus on establishing models with which to identify and test disease-modifying anti-epileptogenic drugs [13].

Hitting a target pharmacologically with a rational or serendipitous therapeutic molecule is the simplest and most high-throughput method of screening and developing new ASMs, and it will no doubt remain extremely important as the focus moves towards disease-modifying agents and treatments for specific types of epilepsy. However, there exists a potentially very useful supplementary approach that pharmacology alone cannot address, namely the utilisation of pharmaceutical formulation. Perhaps the most interesting aspect of this is the potential it offers to exploit endogenous molecules [17,18], which are normally subject to rapid in vivo degradation, but may exert important therapeutic effects where synthetic molecules fail. From another perspective, pharmaceutical formulation might be used to achieve more efficient targeting of drugs to the brain to improve tolerability and efficacy (e.g., through the use of nanoparticles), or for simply incorporating a molecule that is challenging to formulate within a solution or suspension [19,20,21,22,23]. Finally, and perhaps most obvious, it provides a pathway to optimally deliver molecules by non-conventional routes and orifices.

The following review explores the nose, a somewhat alternative approach to ASM delivery, for which pharmaceutical formulation is intimately relevant, and while tapped from some angles, has not yet had its full potential explored. The nose has had longstanding and interesting relationships with both epilepsy and the brain, and this review will discuss these, along with the potential value of delivering ASMs to the brain through the nose as a therapeutic strategy for the treatment of seizures and epilepsy.

## 2. Relationships between the Nose and Epilepsy

### 2.1. Historical and Epidemiological

Historical examples of treating epilepsy through the nose can be drawn from all corners of the world. In the East, the aroma from smelling a shoe has been, and still is, used as a first aid measure to arrest seizures [24]. In the West, the burning of the ammonia-based hartshorn under the nose as a first aid measure for treating seizures was reported in the 17th century [25]. In later times, the use of ammonia or amyl nitrate was thought to arrest the course of a seizure [26], and later still, that such a stimulus may be used to condition a patient to inhibit seizures psychologically by thinking of it during the prodromal phase [27,28]. The commonality between these “treatments” is obviously the potent and disenchanting nature of the aroma. More recently, and in contrast to these reports, Betts proposed conditioning with aromatherapy as a means of controlling seizures. However, while pleasant olfactory sensations may have played a role here, they could not be distinguished from the effects of transdermal oil absorption or simply the relaxation associated with the sessions [29].

Evidence of the involvement of the olfactory sensory network with some types of epilepsies can be found in the symptoms experienced by some patients, namely olfactory auras, and impaired olfactory function [30,31,32]. The usual occurrence in an olfactory aura is the experience of an unpleasant odour, with the earliest influential description of an olfactory case dating back to 1889, describing a woman with a horrible smell of “dirty burning stuff” prior to a seizure [33]. Interestingly, it appears that such auras may be more prevalent in patients with temporal lobe epilepsy, and the involvement of the olfactory network is further supported by the occurrence of many abnormalities in olfactory function in these patients. These include impairment of odour discrimination, memory, and identification, as well as temporarily altered detection thresholds, with an increased sensitivity before a seizure and decreased sensitivity for hours or days afterwards [33]. The fact that temporal lobe epilepsy represents most cases of drug-resistant epilepsy (at least those managed surgically) [34] suggests a potentially significant role for the nose in the treatment of this phenomenon.

### 2.2. Neurological

#### 2.2.1. The Olfactory System

**Figure 1:**
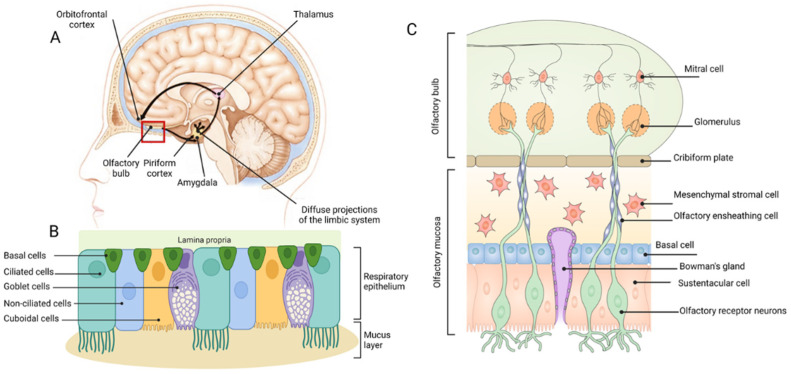
Basic anatomy of the human nasal cavity (**A**), and the respiratory (**B**) and olfactory (**C**) epithelia of the nasal passages. Figure modified from [39]. Image B generated using BioRender^®^.com.

The olfactory epithelium is located in the dorsal or dorsoposterior nasal passage (Figure 1A) and is remarkably similar between different species [35]. It contains bipolar sensory neurons with an axon in the olfactory bulb and a dendrite in the epithelium, capped with numerous cilia that extend into a surface mucous layer and can interact with dissolved odourant molecules (Figure 1C). The axonal synapses of the olfactory neurons converge onto mitral or tufted cells in the glomeruli of the olfactory bulbs [36], with stimulation of different classes of receptor neurons leading to the formation of a map of excited glomeruli [37]. Unlike all other sensory inputs, which are primarily relayed through the thalamus, the olfactory bulbs first transmit signals along the myelinated lateral olfactory tract to project diffusely into the largest region of the primary olfactory cortex called the piriform cortex (Figure 1A), which is only two synapses removed from the outside world [37]. The piriform cortex is made up of three layers: a sparsely populated superficial layer, a main input layer containing densely packed somata of glutamate-releasing principal neurons, and, finally, a deep layer containing principal neurons at a lower density [37]. GABA-releasing interneurons are scattered across all layers and provide feedforward and feedback inhibition of principal cells [37]. From the primary olfactory cortex, information is projected widely to secondary olfactory areas, such as the orbitofrontal cortex via the mediodorsal thalamic nucleus (Figure 1A) [38].

#### 2.2.2. Epilepsy and the Olfactory System

The olfactory system, in particular the piriform cortex, appears to be an important player in epilepsy, as suggested by the historical and epidemiological links described above. Olfactory impairment in some focal epilepsies, especially of the temporal lobe, has been shown by neuroimaging to be associated with changes in the piriform cortex that parallel the odour discrimination, memory, and identification impairment reported [33]. Furthermore, atrophy and reduced olfactory bulb volume have been described [40,41]. Seizures that produce olfactory hallucinations typically show widespread orbitofrontal and anterior temporal lobe activity. Olfactory auras have been suggested to correspond to epileptic activity that causes an intense activation of the piriform cortex and amygdala, as is seen when an unpleasant odour is smelt in the environment [33]. However, it is worth noting that human seizures have been noted to arise from the piriform cortex without an olfactory aura [42]. Interestingly, a similar intense activation of the olfactory cortex is also a hypothesis behind the success of strong odours in the prevention of some seizures by disrupting the synchronised progression of epileptic discharges between regions [33,43]. Alternative explanations include a change in alertness due to a smell, which may interrupt seizure progression, or a pharmacological effect of the odourants [33].

The many neurological connections between the olfactory system and seizures have been reviewed in detail by Vaughan and Jackson [33] and Vismer et al. [44], with both attesting to the great therapeutic potential of targeting the piriform cortex. The links between the two systems are numerous, with implications of a role for the piriform cortex in seizure generation and distribution, epileptogenesis, and pharmacoresistance [33]. As highlighted by Vismer et al. [44], the propagation of seizures through the brain is not a random process and, instead, existing circuits that normally support highly controlled recurrent activity are exploited. In this respect, the anatomical arrangement of neural networks in the piriform cortex make it inherently susceptible to seizure activity [33]. Each glomerulus in the olfactory bulb has over 1000 broad projections (mainly mitral cells) across the piriform cortex to random pyramidal cells [33]. This is necessary to allow detection of complex odour mixtures, but also forms a large, highly interconnected, excitatory network that requires careful regulation by interneurons [33]. If local inhibitory circuits are modified or removed, it is extremely prone to forming hyper-excitable local networks [33]. Furthermore, strong reciprocal connections of the piriform cortex to nearby structures (e.g., olfactory bulbs, amygdala, hippocampus) normally provide an additional means of modulating olfactory inputs, but run the risk of becoming circuits that could sustain seizure activity [33,45].

Vaughan and Jackson [33] reviewed the roles of the piriform cortex in the generation and distribution of seizures. In terms of generation, the most obvious connection can be found in the deep anterior piriform cortex, which contains a well-known chemoconvulsant trigger zone called the ‘area tempestas’ [44] that is crucial for seizure initiation within the limbic network. In addition to chemical stimulation, the piriform cortex can be electrically kindled to generate seizures that follow the same progression of motor features as kindling from other sites, such as the amygdala [33]. With regards to the distribution of seizure activity, the authors noted the role of the piriform cortex in the process of amygdala kindling [45,46,47], as well as the loss of GABA-ergic interneurons in it during this process [48]. The authors also noted the piriform cortex’s role as a common target of discharge spread in frontal and temporal lobe epilepsies, indicated by the sites of lesions that can produce an olfactory aura [31], the impact of these epilepsies on olfactory function [49], and detection of piriform cortex activity by EEG-fMRI [50]. The relationship of the piriform cortex to clinical descriptions of aura progression and its broad outputs to cortical and subcortical regions were also discussed. Both this distributive ability, combined with the potential for sustained hyper-excitability, form the basis of hypotheses for a role of the piriform cortex in epileptogenesis (through recruitment as a secondary hyper-excitable node) and drug resistance (through alterations in neural networks) [33]. Though these processes fall outside the scope of this review, it is interesting to consider the effect piriform cortex-targeted therapeutics may have on them in light of these theories.

#### 2.2.3. Clinical and Social

Despite the extensive aforementioned neurological links between the olfactory sensory network and seizures, the current clinical application of intranasal treatments for seizures have been birthed from an entirely different opportunity: the rich vascular bed present in the lower nasal passage [51]. The nasal vascular bed is an ideal site for rapid absorption of lipophilic therapeutics, namely the benzodiazepines, which have so far been used for this purpose. Intranasal administration has proven valuable in addressing the need for a practical, effective, and socially acceptable treatment for seizure emergencies outside of hospital, including prolonged single seizures, acute repetitive seizures, and status epilepticus [52]. Prompt treatment for seizure emergencies occurring outside of hospital has been shown to reduce the risks associated with progression to status epilepticus [53].

The most studied benzodiazepine for intranasal administration is midazolam, which was first reported to treat acute seizures in 1996 [54] and was followed by a number of other studies testifying to its efficacy and safety [55,56,57,58,59]. Intranasal administration achieves 67 to 100% bioavailability and peaks within 10 min, leading to seizure control within 2 to 5 min after administration [60]. A nasal spray formulation of midazolam (Nayzilam^®^) was recently approved by the Food Drug Administration (FDA) for short-term treatment of seizure clusters in patients aged 12 years or older [61]. Diazepam, the intravenous benzodiazepine of choice (half-life around 50 h) and the rectal competitor of intranasal midazolam, has also been tested by the intranasal route. Peak plasma concentrations are reached significantly later than with intranasal midazolam (45 min vs. 10 min [62]), but time to onset of seizure cessation has not yet been reported, and neither has a head-to-head trial with midazolam. It is unclear why peak plasma concentrations of diazepam are achieved later then midazolam as both molecules have similar lipophilicity. It is likely that different formulation compositions may influence intranasal bioavailability, leading to this observed difference [63]. Like midazolam, a nasal spray (Valtoco^®^) of diazepam was also approved by the FDA in early 2020 for short-term treatment of seizure clusters in patients aged 6 years or older [64]. Finally, lorazepam is reported to be 4 to 6 times less lipid soluble than midazolam and diazepam, and it has been found to have a peak effect time of 30 min and a half-life of 18.5 h after intranasal administration [65,66]. Intranasal lorazepam has been evaluated in studies in children and found to be comparable to intravenous lorazepam, with the same median onset time [67,68]. However, it is uncertain whether any benefit is obtained from lorazepam compared with midazolam, other than perhaps having an extended duration of action and being easier to formulate than diazepam.

Overall, intranasal administration of anti-seizure medication is a rapid, effective, and socially acceptable practice with industry engagement in product development, both in terms of formulation and administration devices [69]. However, the scope is currently limited to benzodiazepines, which are really only an emergency treatment for severe prolonged or cluster seizures. It should also be noted that because systemic absorption is the main proposed route of entry into the brain, high doses are still required; thus, it does not offer any benefit in terms of decreasing systemic exposure. The field of pharmaceutical sciences has been increasingly exploring the potential of direct nose-to-brain transport pathways to address challenges in the clinical treatment of seizures and epilepsy.

## 3. The Anatomy and Physiology of Intranasal Administration to the Brain

### 3.1. The Nasal Passage and Epithelia

The nasal septum divides the nasal cavity longitudinally into two passages, each having three key regions: the nasal vestibule, the respiratory region, and the olfactory region [36,70]. The latter two comprise the main chamber of the nasal passage and essentially consist of an epithelial layer covered by a continuous layer of mucous. Bony structures (turbinates) lined with mucosal tissue project into the lumen to increase the surface area of the nasal passage and facilitate filtering, humidification, and warming of inspired air [70]. Four types of epithelia exist in the nasal passages and help distinguish the different regions. The nasal vestibule primarily contains a squamous epithelium, which becomes a non-ciliated, cuboidal/columnar (transitional) epithelium, then a ciliated, pseudostratified cuboidal/columnar (respiratory) epithelium in the anterior main chamber, and, finally, the olfactory epithelium in the dorsal or dorsoposterior main chamber [70]. The respiratory and olfactory epithelia shown in Figure 1B and 1C, respectively, will be the focus of the following discussion as they are the most relevant to therapeutic delivery to the brain [40]. For detailed reviews of nasal anatomy to supplement the following text, the reader is referred to more extensive reviews [36,70,71,72,73].

### 3.2. Respiratory Epithelium

The respiratory epithelium (Figure 1B) consists of goblet cells, cuboidal cells, brush cells, basal cells, and ciliated and non-ciliated columnar cells [36,74]. It also contains various glands for producing nasal secretions, in addition to the mucous secreted by goblet cells [36]. The mucous layer consists of a low viscosity layer, which bathes the cilia, and a more viscous layer on top [75]. Deposited substances are generally subject to rapid mucociliary clearance by the motile cilia of the brush cells, which results in removal from this region in approximately 15 to 20 min [72]. The respiratory epithelium has a far richer supply of blood vessels and lymphatics in comparison to the olfactory epithelium [73]. Interestingly, it is innervated by branches of the trigeminal nerve, many fibres of which extend through the epithelium so that their free nerve endings lie just beneath tight junctions (i.e., very close to the surface) [36]. The trigeminal nerve has a predominantly sensory function, whereby information, in the case of the nasal epithelium fibres, is relayed back to both the brainstem at the level of the pons and a small portion to the olfactory bulbs [36,73]. It should be noted that while most significant to the respiratory epithelium, the extension of free trigeminal nerve endings to near the surface is also a feature of the olfactory epithelium [36].

### 3.3. Olfactory Epithelium

The key feature of the olfactory epithelium is the many dendrites of bipolar sensory (olfactory) neurons extending out from the CNS to make direct contact with the external environment [75] (Figure 1C). Each dendritic process ends in a small swelling, known as the olfactory knob, which projects 10 to 23 cilia into the overlying mucous layer [75]. It is important to note that in contrast to the respiratory epithelium, these cilia are non-motile; hence, dynamic mucociliary clearance does not occur in this area [36,70,75]. Rather, mucous slowly drains into the respiratory region when it is over-produced. The axons of each olfactory neuron are collected into nerve bundles surrounded by interlocking olfactory ensheathing cells (the fila olfactoria), which are subsequently collected into a bunch of nerve bundles and further enclosed by fibroblasts to form a peri-neural sheath [73]. These channels extend back through small gaps in the cribriform plate (separating the nose and the cranial cavity) to enter the cranial cavity, pass through the subarachnoid space containing cerebrospinal fluid (CSF), and synapse (along with around 1500 other olfactory neuron axons) with a single mitral of tufted cell in the olfactory bulb [75]. Other features of the olfactory epithelium include microvillus sustentacular cells, which act as adjacent supporting cells for the olfactory neurons [36]); Bowman’s gland cells, which form ducts originating in the lamina propria and produce a serous fluid to aid dissolution of odourant molecules [36]; and horizontal basal cells, which lie along the basal lamina and act as progenitors to olfactory neuron progenitor basal cells, sustentacular cells, and cells of the Bowman’s gland and duct. As in the respiratory epithelium, blood and lymphatic vessels also exist in the lamina propria [36], but to a lesser extent [73].

## 4. Nasal Routes of Absorption for Therapeutics

**Figure 2:**
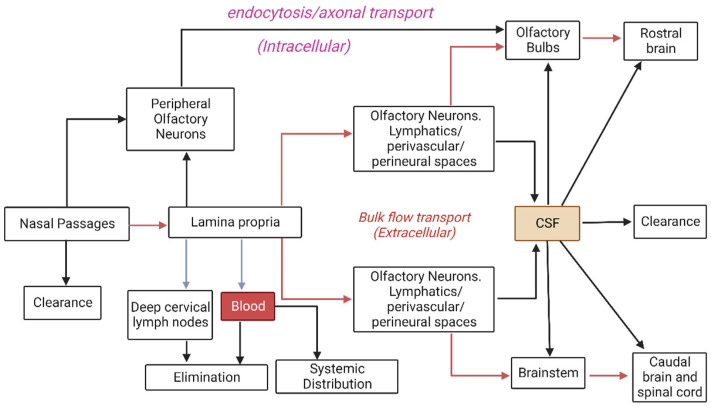
Pathways by which intranasally-administered therapeutics may be cleared or transported to the brain. Figure modified from Lochhead & Thorne. [36] (Created with Biorender^®^.com).

The features of the abovementioned epithelia provide a number of potential delivery routes to the CNS, collectively divided into the olfactory and respiratory pathways. These have been reviewed in detail elsewhere [36,73,76,77], but will be summarised below. As detailed in Figure 2, the different pathways are most easily classified as systemic, intracellular, or extracellular, and a prerequisite for all pathways, other than intracellular transport via olfactory neurons, is an initial transport into the lamina propria. Depending on the properties of the molecule, macromolecule, or particle/delivery system concerned, it may achieve this via paracellular transport through tight junctions or, alternatively, passive diffusion or transcytosis through epithelial cells. Alternatively, it will be trapped in the nasal mucous and eventually cleared from the surface.

### 4.1. Systemic Transport

As indicated previously, the nasal mucosa is highly vascular, which can lead to extensive, and possibly undesired, systemic absorption of therapeutics, especially via the more endowed respiratory epithelium. Vasculature in this region has a mixture of continuous and fenestrated endothelia, permitting transport of both small and large molecules into the systemic circulation [73]. A proposed advantage of systemic intranasal delivery into the bloodstream may be the potential for ‘counter-current transfer’, whereby drugs may enter the venous blood supply in the nasal passages, but then be rapidly transferred to carotid arterial blood, thereby reaching the brain rapidly and in higher concentrations compared to if it underwent an initial distribution throughout the systemic circulation [73]. Alternatively, if substances are not absorbed into the bloodstream, they may drain into the lymphatic vessels in the lamina propria and ultimately to the cervical lymph nodes [36].

### 4.2. Intracellular Transport

The holy grail of brain delivery pathways is the intracellular transport of therapeutic molecules directly through the olfactory neurons. Given that these neurons extend numerous cilia directly into the mucous covering the surface of the epithelium, providing a large surface area for odourant detection, the hope has been that they may also provide a large surface area for therapeutic absorption by passive diffusion, or in the case of larger macromolecules or nanoparticles, a receptor-mediated or adsorptive endocytosis. Therapeutics might then diffuse or be transported as cargo through these neurons directly to their axonal synapses within the CNS [71]. Studies have shown that large molecules—such as horseradish peroxidase, wheat germ agglutinin-horseradish peroxidase, and albumin, as well as some viruses—may be transported intracellularly along the olfactory neuron axons towards the brain [36,78]. A similar route has been proposed for intracellular transport through trigeminal nerve fibres [36]; however, this would first require transport of the molecules into the lamina propria via other pathways, as discussed earlier. Given that the trigeminal nerve transmits information to both the brainstem and olfactory bulbs, albeit to varying degrees, it can be difficult to infer from experimental data the route(s) (trigeminal or olfactory) by which intranasally administered molecules reach the olfactory bulbs if they appear in both regions [73]. Despite the apparent potential for intracellular delivery through these neurons, the current consensus seems to be that this pathway is too slow to mediate the rapid direct uptake of the various molecules reported in the literature, which is instead attributed to extracellular pathways [36,72,73]. It may therefore have limited relevance in acute nasal delivery applications.

### 4.3. Extracellular Transport

Extracellular pathways from the nose to the brain are believed to play the most significant role in rapid and direct transport of molecules into the CNS [36]. They primarily involve bulk flow, by extracellular diffusion or convection in perineural or perivascular spaces associated with nerve bundles or blood vessels, passing through the cribriform plate to the olfactory bulbs or the anterior lacerated foramen to the brainstem [36]. The perineural spaces surrounding the olfactory and trigeminal nerves appear to allow transport of some molecules into the subarachnoid space [73,79]. It has been suggested that this may be facilitated by the propulsion of molecules by structural changes occurring during depolarisation and propagation of action potentials in adjacent axons in the fila olfactoria [73,80]. Similarly, between the outermost layer of blood vessels and the basement membrane of surrounding tissue exist perivascular spaces, through which bulk flow is thought to be facilitated by arterial pulsations. Interestingly, it has been suggested that if molecules can exploit such a pathway to travel into the CNS, movement deeper into the brain via a cerebral perivascular network or CSF flow pathways could result in rapid and widespread distribution [36,81,82].

Lochhead and Thorne [36] proposed that in order to exploit bulk flow pathways, a molecule would need to reach the lamina propria (e.g., via paracellular transport) and escape absorption into blood vessels and drainage into lymphatic vessels. However, they also noted the interesting possibility that molecules might be able to move more easily into such bulk flow pathways, on the basis that olfactory neurons are constantly regenerating (about every three to four weeks [73]) and olfactory ensheathing cells maintain continuous open spaces for the regrowth of new fibres during this process. It should be noted that the key role of perivascular and perineural channels is to drain neuronal waste from interstitial fluid, thus net flow is believed to be away from the CNS [73]. However, it has been proposed that flow could be bidirectional, depending on such factors as posture and local vessel architecture [36,81], and the existing literature seems to support this, given the data suggesting intranasally administered molecules are able to rapidly move into the CNS via this route.

## 5. Animal Models for Intranasal Delivery

The rat is the most commonly used model for studying direct nose-to-brain delivery routes [83]. Rats have a similar nasal epithelium, submucosa, and olfactory sensation network to humans [37,70,83], and are a relatively cost-effective and easy-to-handle model [83]. They do, however, exhibit some important anatomical and physiological differences that must be kept in mind when considering the potential for extrapolation of experimental results to humans. These parameters have been reviewed by others [73,75,84] and are summarised in Table 1, but the two most significant will be briefly discussed below.

Commonly referenced is the relatively small proportion of the total nasal epithelium that constitutes the olfactory region in humans compared to rats. This may be expected to have a large impact on the percentage of drug transport via direct olfactory pathways (as opposed to respiratory epithelium-associated systemic pathways) between rats and humans. Selective deposition of drugs on the olfactory epithelium may act as the first step towards addressing this issue, but the actual surface area available for absorption and the size of the olfactory bulbs that drugs may be transported to must also be considered. On an absolute scale, the olfactory bulbs of humans are larger than those of rats [85]. Traditionally quoted values of olfactory epithelium surface area in humans (e.g., 5 cm^2^ vs. 6.75 cm^2^ in rats) [75,86] have therefore implied that a higher percentage of drug delivery might be required from a smaller olfactory surface area to achieve comparable olfactory bulb concentrations. More recent reviews, however, have suggested that the olfactory epithelium of humans constitutes a larger area (e.g., 12.5 cm^2^) [22,36,86], which puts the absolute surface area at almost double that of rats and tips in favour of the theoretical translatability of drug delivery through this region.

The second important factor to consider concerns cerebrospinal fluid (CSF). As indicated earlier, current literature suggests that the most rapid and significant direct route from the nose to the brain may be in the CSF through perineural or perivascular channels. Given that the volume of CSF in humans is much greater than that of rats [75], a drug may undergo significant dilution if it is widely dispersed by this pathway in the brain. This suggests that brain concentrations detected in rat models may significantly overestimate those which would be expected in humans. However, drugs in the CSF would be expected to come into contact with the olfactory bulbs first, and preferential transport (e.g., diffusion) into the parenchyma here due to the high concentration gradient may still permit sufficient targeted delivery. Furthermore, CSF turnover rate of rats (hourly) is higher than in humans (5 hourly) [75], which suggests that if a drug reaches the brain via the CSF in humans, it will have a longer period to cross into the parenchyma than in the rat.

## 6. Animals as Seizure and Epilepsy Models for the Evaluation of Anti-Seizure Therapeutics

### 6.1. Overview of Key Models

To screen for the anti-seizure activity of a compound, simple, high-throughput models are preferred to avoid investing extensive time and resources on inactive compounds [9]. Many models of seizures and epilepsy have been described [15,87]; however, the problem with most is that they have not been clinically validated [87], i.e., shown the ability to correctly predict the effectiveness of a drug in humans. Traditionally, the Anticonvulsant Screening Programme (ASP), recently rebranded the Epilepsy Therapy Screening Programme (ETSP), has used the Maximal Electroshock Seizure (MES) test and the s.c. PTZ test for this purpose due to their simplicity and good predictive value for clinical efficacy in humans [15]. Another simple test, the 6-Hz test, has made its way into the acute screening protocol in more recent times to identify therapies that may be effective against “drug-resistant” seizures. Kindling has also been used as a validated chronic model to secondarily differentiate effectiveness in partial epilepsy.

For any seizure experiment, it is essential to consider the hypothesis that is being tested when selecting a model [87]. In the context of this review, it is the hypothesis that intranasal delivery of ASMs will elicit anti-seizure effects by way of one or more of the pathways discussed earlier. Therefore, as the most validated and commonly used models of primary ASM testing, the above-mentioned four models (as well as a variation of MES, the MEST) will be described below, followed by a discussion of their usefulness in the context of assessing intranasal therapies.

#### 6.1.1. Maximal Electroshock Seizure Test

**Figure 3:**
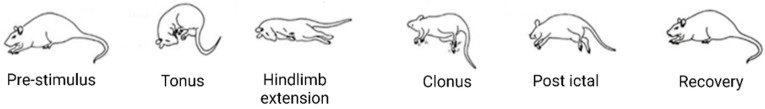
Typical stages of MES seizures.

The Maximal Electroshock Seizure (MES) test was the first model to be used to systematically screen compounds for anti-seizure efficacy, leading to the discovery of phenytoin in 1937 [88]. The test is considered a measure of the effect of a drug to prevent seizure spread through neural tissue and, thereby, prevent generalised tonic-clonic seizures [87]. The classic procedure entails the application of a suprathreshold electrical stimulus to mice (50 mA) or rats (150 mA) using a constant current stimulator with a sinusoidal alternating current waveform for 0.2 s at a frequency of 50 to 60 Hz [87,89]. The endpoint is usually tonic hind limb extension (HLE), the most severe seizure behaviour (Figure 3) resulting from this type of stimulation. Naïve animals are pre-tested to ensure they exhibit this behaviour, and failure to demonstrate tonic HLE on a subsequent stimulation after treatment implies anti-seizure drug action. The stimulus is commonly applied through corneal electrodes, but auricular electrodes may also be used. While initially thought to be equivalent, studies have shown differences in the characteristics of seizures elicited by each of these mechanisms. For instance, transauricular seizures have been shown to produce tonic HLE more reliably at maximal currents, as well as to decrease latency to and increase duration of HLE [90,91]. It is a simple procedure in that the outcome is binary and the suprathreshold nature of the stimulus reduces variability in response, but it does run the risk of failing to detect more subtle anti-seizure effects, such as is the case for primidone and clonazepam, which are known to be clinically effective in humans, but which produce a negative result [91].

#### 6.1.2. Maximal Electroshock Seizure Threshold Test

The MEST test is a measure of the effect of a compound on seizure threshold, rather than spread, and is therefore more sensitive to detecting anti-seizure hits. For example, as mentioned above, primidone and clonazepam do not show anti-convulsant activity in MES. However, a dose-dependent anti-convulsant effect is detected by MEST, which translates to humans [91]. The aim of this test is to determine the current that elicits tonic HLE in 50% of a group of animals (i.e., CC_50_—the convulsive current in 50% of animals) [92]. This is commonly determined using the “up and down” method of Kimball et al. and involves stimulation of a group of animals in series, where the current used for stimulation of a given animal depends on the response of the preceding animal [16,91,93]. If the preceding animal displays tonic HLE after stimulation, the current for stimulation of the next animal is lowered, usually by 0.06 log units in rats and 0.01 log units in mice [91]. If the preceding animal did not display HLE, the stimulation current is elevated by the same log interval. The current used for the first animal is determined by the researcher but must approximate the CC_50_ of the group [93], which can pose some technical difficulty with the use of this model. The complete data set of responses is used to calculate the CC_50_ for the group. One advantage of this model is that animals can be subjected to multiple stimulations, as the threshold does not significantly change, provided at least 48 h is left between sessions [94]. In this way, control and treated thresholds can be assessed in the same group of animals to lessen variability. In contrast to MES, this test will also yield information on pro-convulsant effects if these are present [95].

Despite the potential usefulness of the MEST test in more sensitively screening for anti-seizure (or pro-seizure) effects [91], it has not been adapted by the ETSP. This is most likely due to a combination of reasons that make it more technically complex and variable, and, therefore, decrease throughput and increase cost. Aside from these drawbacks, the other major limitation is that it gives no insight into the mechanism of action of the compound, i.e., whether it elevates threshold or prevents seizure spread or both [96].

#### 6.1.3. Pentylenetetrazole Test

The aim of this test has classically been to find the convulsive dose of subcutaneously injected PTZ inducing a clonic threshold seizure of at least 5 s duration in 97% of animals (CD_97_) by observing animals for a post-injection period of 30 min for such a “threshold” seizure, after which they are euthanized [87]. Part of the problem with the PTZ test has been its dependence on pharmacological actions to produce acute seizure behaviours. This requires consideration of route, doses, metabolism, time of measurement, and pharmacokinetics and pharmacodynamics of the test drug, as well as interspecies variation in all these things, and has led to conflicting data for some drugs between labs, a subject that has been discussed in detail elsewhere [92]. Traditionally, it has been a s.c. injection, but intravenous administration has been suggested as an alternative to overcome some of the limitations associated with PTZ delivery by the s.c. (or even the intraperitoneal) route. Key issues with the model include interspecies variation in the metabolism of PTZ, and use of the model for the analysis of drugs with a short duration of action that peaks early in the 30 min observation period and has necessitated the use of ‘time to seizure onset’ as the measure of effectiveness in many studies [92]. Time to the first threshold seizure (after s.c. injection) or initial myoclonic twitch (during intravenous infusion) therefore appear to be the most reliable endpoints to differentiate ASMs [92]. Being a threshold test, seizure behaviour as a whole may also be assessed in order to provide a more sensitive measure of anti-seizure effect and enrich the prediction of possible clinical potency against different seizure types [92]. Finally, U-shaped dose curves have been reported to contribute to variability with some drugs (e.g., phenytoin and carbamazepine) due to possible pro-convulsant effects induced at high doses in rodents and humans, thus testing at a single high dose is not recommended.

#### 6.1.4. 6-Hz “Psychomotor” Seizure Test

The 6-Hz test was first developed in the 1950s, but at the time was largely disregarded due to a lack of response to phenytoin, which was interpreted as a poor utility to predict efficacy in humans [97]. More recent times have seen its resurrection [98,99] and ultimately elevation to the ETSP testing pathway as an acute model of “drug-resistant” partial seizures [9]. The endpoint of the test is described as immobility or stun, awkward but upright posture, Straub (elevated) tail, facial automatisms (head nodding, jaw movement, twitching of the vibrissae), and forelimb clonus [98,100,101]. It is induced by means of corneal electrodes, through which a rectangular pulse train with a frequency of 6 Hz and pulse width of 0.2 ms is passed for 3 s [98]. Two currents are conventionally used, 32 mA and 44 mA, corresponding to the 1.5 × CC_97_ (current at which 97% of animals demonstrate the endpoint), and 2 × CC_97_, reported by Barton et al. in their characterisation of the model [98].

The 6-Hz model had previously only been characterised in mice [98,99]; however, its very recent pharmacological characterisation in rats [100] stands to expand its potential scope as an acute screening model for activity in “drug resistant” seizures. While the 6-Hz test is a relatively simple model of great interest for the modern screening of compounds for activity in pharmacoresistant epilepsy, the model appears to require further characterisation and development to realise its full potential. It should be noted that demonstration of the efficacy of levetiracetam in the 6-Hz model was done retrospectively, and despite currently being used by the ETSP to differentiate compounds, it has also yet to earn its clinical usefulness. For example, several investigational ASMs (brivaracetam, carisbmate and retigabine) have potently suppressed 6-Hz seizures at 44 mA, but have not shown evidence of effectiveness in humans with drug-resistant partial seizures [102].

#### 6.1.5. Kindling

Kindling traditionally involves repeated excitatory electrical stimuli via a depth electrode surgically implanted into a region of the limbic system (for example, the amygdala, which will be discussed in this review given its relevance to temporal lobe epilepsy). This induces partial, and later secondarily generalised, seizures that increase in length and severity with continued stimulations, ultimately creating an animal with a permanently increased susceptibility to seizures. Seizure severity is classified according to the Racine scale [103,104,105].

Initially, the threshold for inducing after-discharges (the after-discharge threshold (ADT)) is determined by a stepwise procedure, then constant current stimulations are delivered once daily through the electrode until this induces reproducible (e.g., at least 10), fully kindled, secondarily generalised seizures (i.e., Stage 5 on the Racine scale) [103,105]. The ADT is then determined again in the kindled animal on multiple occasions until this too is reproducible [103,105]. Recorded parameters include seizure severity, seizure duration, after-discharge duration (ADD), and generalised seizure threshold (where this differs from ADT), which are defined elsewhere [103,105]. The effect of treatments or other variables on kindling development can also be evaluated by comparing the number of days until the first Stage 5 seizure, the number of days until the fully kindled state is reached, the cumulative seizure duration, and the cumulative ADD [103,105].

In contrast to models of acute seizures, kindling is a model of chronic epilepsy and, therefore, is thought to represent the epileptic brain much better when testing anti-seizure interventions [87]. The changes that occur in the brain as a result of limbic kindling have been linked to those that occur in human temporal lobe epilepsy [104], and it is the only model that has successfully predicted (i.e., not retrospectively) the clinical usefulness of novel ASMs, such as levetiracetam, against partial seizures in humans with epilepsy [15]. Furthermore, models of pharmacological resistance have been developed from it [105,106,107], such as the phenytoin-resistant rat, which provide scope for assessing the ability of new treatments to overcome certain mechanisms of resistance (e.g., the multi-drug transporter hypothesis) [108]. However, limbic kindling is a very labour-intensive and time-consuming process, making it unsuitable as an initial screening model. Potential replacements (e.g., corneal kindling), however, have so far been unsuccessful as their predictive ability is not clear [15,87].

### 6.2. Relevance to the Evaluation of Intranasal Delivery Pathways

As discussed earlier, there are three main pathways that intranasal therapeutics are thought to exploit to reach the brain: the direct olfactory, direct trigeminal, and indirect systemic pathways. Based on previous reports and theoretical considerations, these may target drugs to the olfactory bulbs and piriform cortex, the brainstem, or the whole brain via blood vessels, respectively.

Considering the olfactory pathway first, an ideal model would exhibit focal seizures generated or propagating through the piriform cortex or closely associated areas, such as the amygdala. The most obvious, therefore, would be the very well-characterised amygdala kindling model of epilepsy, in which seizures secondarily generalise from this region. Though the nose-to-brain field is still, as Kozlovskaya [84] puts it, immature, perhaps the most well thought out publications (in terms of marrying hypothesis to method selection) in the current intranasal ASM delivery literature [22,23] have employed this technique somewhat successfully, as will be discussed in the next section. In contrast, a model described above that does not appear to have been used before, but reportedly represents acute focal seizures in the relevant regions [98], is the 6-Hz seizure test. As discussed, while it appears to still be a model in need of more reproducible and thorough characterisation, its recent expansion to the rat arena makes it an intriguing potential platform for assessment of olfactory delivery, particularly in light of its technical simplicity relative to the kindling model.

In the MES model of generalised seizures, the olfactory targeting pathway would seem to have minimal relevance, given that it functions in the forebrain, whereas it has been shown, by way of precollicular lesions, that the tonic components of corneal MES seizures do not depend on the forebrain for their initiation or progression [95]. In further support of this, the “area tempestas”, part of the piriform cortex that is very sensitive to induction of seizures by GABA antagonists and is thought to function as a broadcasting system by triggering generalised seizures in response to stimulation of limbic circuits, cannot exert control over tonic seizures induced by corneal MES. Again supporting that generation of these seizures does not depend on the forebrain [109]. Therefore, any selective forebrain delivery of drug to areas like the piriform cortex would be expected to be ineffective in stopping the spread of corneal MES seizures. However, a potential effect on the clonic components, despite not being the endpoint of this test, cannot be ruled out. As MEST similarly uses tonic endpoints, it would also likely be of little use. Minimal electroshock threshold could be considered, but is not a preferred test for reasons discussed above. Likewise, one might also consider the use of minimal seizures observed in the PTZ model, but given its systemic, pharmacological nature, it would be expected to have widespread effects throughout the brain, suggesting that it would lack the specificity required to assess intranasal delivery in targeting a focus and the spread of seizure activity. Transauricular MES (and MEST) seizures would seem to have even less involvement than transcorneal MES with the forebrain and given that lesions of the amygdala have no effect on transauricular electroshock seizures (or tonic audiogenic seizures for that matter) [109], a drug effect targeted to this region is unlikely to be detected by this model. A clonic phase is reportedly not reliably seen after the tonic phase resulting from transauricular stimulation [89], so may not be a suitable alternative for assessment. Despite the predicted failure of this test to model olfactory pathway delivery to the limbic regions, its role in detecting trigeminal pathway delivery to the brainstem would seem a lot more promising, as will be discussed shortly.

In order to speculate about the possible effects of inhibitory drugs being focally delivered to olfactory networks, it is interesting to consider a study which reported the effects of olfactory bulb ablation in mice on response to seizure tests [110]. In corneal MES, olfactory bulb ablation was reported to decrease the duration of clonic convulsions and postictal coma; however, it did not affect the tonic component, which is consistent with the above discussion. In contrast to the predictions above, the authors found a marked increase in corneal electroshock seizure threshold after ablation of the olfactory bulbs. It should be noted that although the authors reported using a minimal electroshock threshold test (defined by Swinyard [101] as clonic activity of the vibrissae, lower jaw, or forelimbs, without loss of posture), they classified a “minimal full seizure” as including running movements, clonic convulsion, tonic flexion, and tonic extension. This would suggest that their measure was closer to the definition of the threshold for a maximal electroshock seizure, although it was not specified whether the flexion and extension involved the hindlimbs. The CC_50_ reported for their controls is similar to the maximal electroshock seizure threshold reported elsewhere for CF-1 mice (8.85 mA) [96], which suggests this was the likely measure reported. Finally, in the s.c. PTZ test, total incidence of convulsion was not different from control; but in contrast to the MES model, clonus was more marked and long-lasting in mice without olfactory bulbs, suggesting a decreased inhibition of seizures by the olfactory bulbs. Such a theory may be consistent with the ability of strong olfactory stimuli to interfere with kindled seizures [42], but it introduces uncertainty as to the effect inhibitory ASMs would be expected to have in the PTZ model. To add to the complexity, tonic convulsion incidence was decreased, indicating an apparently opposite effect of olfactory bulb ablation on this component. Overall, the effects of olfactory bulb ablation are a lot more complex and far-reaching than might be expected from acute drug administration, but they offer insight into the role of the olfactory networks in these seizure models.

The trigeminal pathway primarily offers a potential route from the respiratory mucosa through the back door of the brain via the brainstem, and assessment of drug delivery by this route calls for a different approach from a model. As stated earlier, the MES and MEST tests present as the most obvious candidates, given their unquestionable relationship with the brainstem. Transauricular stimulation may possibly have advantages over transcorneal in that it would appear to generate seizures directly through the brainstem, rather than initially spreading through the frontal brain, providing a more specific assessment of focal drug delivery. Of note, it has been used in a few studies on this topic [111,112,113], which will be discussed in the next section. One can conclude that MEST presents itself as a more appropriate initial candidate for assessment of intranasal delivery to the brainstem to detect an effect on seizure threshold, rather than setting the bar as the ability to interfere with a superthreshold stimulus that runs the risk of masking more subtle information. For reasons already discussed, the kindling and 6-Hz models would likely be of little use in assessing delivery by this pathway, while the PTZ model again may suffer from eliciting a pharmacological effect on the whole brain.

Finally, for assessment of drug delivery by a systemic pathway (or an alternative widespread brain delivery pathway, such as a direct transport in the CSF), any model may feasibly detect an anti-seizure effect of intranasally delivered drug. This will, however, be largely dependent on the usual ability of the model to detect a specific compound after systemic administration (e.g., phenytoin is reported to be effective in MES, MEST, and kindling, but generally not in the PTZ or 6-Hz models), as well as the dose of drug that is able to be delivered through the nose, which in the context of the systemic circulation will be subject to the usual impeding factors (e.g., dilution, protein binding, metabolism, efflux transporters), and may mean that the relatively low doses attainable through the intranasal route could render them completely ineffective. Nonetheless, intranasal pharmaceutical studies with ASMs to date have generally reported a direct nose-to-brain delivery component co-existing with a significant systemic component, but still with some degree of anti-seizure efficacy where this was tested. The following section will discuss these studies and what can be learnt from them as a whole to move forward.

## 7. Pharmaceutical Formulation of Anti-Seizure Therapeutics

### 7.1. Role of Pharmaceutical Formulation

The potential advances from exploiting a direct nose-to-brain delivery route to deliver anti-seizure therapeutics, as well as the essential role of pharmaceutical formulation in achieving this, has been discussed earlier in the review. The clearest advantage of direct delivery would be the avoidance of the systemic circulation, at least prior to initial contact with the brain. The doses of ASMs required to achieve therapeutic plasma concentrations are much larger than the quantities of drug that are required in the brain [114,115], secondary to pharmacokinetic factors, such as systemic metabolism, plasma protein-binding, clearance, and widespread tissue distribution. A direct intranasal route may, therefore, allow the administration of much lower doses to increase tolerability, a modifiable contributor to the definition of drug-resistant epilepsy. Furthermore, while still speculative, a direct delivery by an olfactory pathway to seizure generating or propagating regions, such as the piriform cortex, may also play a role in circumventing proposed mechanisms of resistance, such as inadequate drug levels reaching these regions due to overactive efflux transporters at the blood–brain barrier [116,117]. Even in responsive epilepsy, such a pathway might conceivably be exploited as a means of controlling some types of focal epilepsy and not just reducing systemic exposure, but also exposure of unproblematic brain regions to the drug. To test all these blue-sky visions, however, there are challenges to overcome, both in therapeutic formulation (reviewed elsewhere [22,23]) and the pre-clinical evaluation of the mechanisms by which direct nose-to-brain delivery may be an effective therapeutic tool. While research into the latter expands beyond just anti-seizure treatment, the following section will be limited to discussing studies which explore this therapeutic use.

### 7.2. Studies of Pharmaceutical Formulation for Anti-Seizure Therapeutic Delivery

Anti-seizure therapeutics have been formulated into a range of different pharmaceutical delivery systems to date in attempts to exploit a direct nose-to-brain delivery (Table 2). The components of these formulations are listed for the reader’s reference, given that administration vehicles can potentially confound the results of seizure tests [118], but the specific formulation methodology, ingredient rationale, stability, and release properties fall outside the scope of this review. Instead, it will discuss methodological aspects of in vivo testing of such formulations and what can be learnt from the endeavours to do so thus far. The discussion should be taken in the context of a recent review of intranasal pharmaceutical formulations in general that suggested that compounds reach the brain most efficiently by direct routes in the order of particles > gels > solutions, but in terms of total brain delivery, the order was gels > particles > solutions, suggesting a higher systemic contribution from gels [84]. However, it should be noted that the authors did not differentiate whether particles referred to nanoparticles, microparticles, or both, which is important as these systems, reviews of which can be found elsewhere [71,119,120], may act differently to deliver drugs.

#### 7.2.1. Administration Technique

Several techniques have been used for intranasal administration to rats over the years. Most studies have been carried out in anaesthetised rats in a supine position to facilitate deposition and retention on the olfactory epithelium, which comprises the upper third of the nasal cavity [73]. Most researchers position animals with their head horizontal to the bench to prevent drainage to the oesophagus and trachea when supine [73]. Historical techniques have involved cannulation of the trachea to aid breathing, then circulation of drug solution in the nasal cavity by a peristaltic pump under anaesthesia or alternatively, sealing the oesophagus with adhesive before administration (and possibly the nares after administration) of a small volume with a micropipette [83]. In contrast, more modern approaches involve administration of a small volume through the nares to the conscious or lightly sedated rat [83]. This may be a single dose administered to one nostril via insertion of polyethylene tubing [121], or smaller aliquots gradually sniffed in over a period of time after placement on the nares [122]. The former offers the advantage of being able to direct the dose to the posterior nasal cavity where the olfactory epithelium lies, in contrast to the latter, which will have significant initial contact with the respiratory epithelium and consequently be subject to rapid mucociliary clearance and higher systemic absorption. Tubing-mediated delivery may also be used simply to ensure adequate coverage of the nasal mucosa via a deep delivery point. In line with these advantages, Table 3 shows that most studies investigating intranasal delivery of ASM formulations employed a similar tubing administration method. Interestingly, Czapp et al. [123] reported alternating between delivery at the opening of the nares and tubing-mediated delivery into the deep nasal cavity, which may have had implications for their pharmacokinetic and efficacy results, which did not differentiate between the two administration techniques.

The volume of an administered dose may affect deposition within the nasal cavity and is a key challenge to the intranasal delivery of lipophilic therapeutics, such as most ASMs, in general. There is a balance between adequately covering the epithelia through which absorption is intended (olfactory and/or respiratory) and avoiding so large a volume that it overflows out of the nasal passage, causing a lower dose to be delivered and running the risk of deposition in the nasopharynx and subsequent inhalation causing respiratory distress in an experimental animal [73]. Rats generally receive a volume of 40 to 100 µL if given as a series of drops applied to the nares, whereas with administration to the posterior nasal passage via tubing, lower volumes of 20 to 40 µL are used, as there is less surface area to cover [73]. Considering this, the rationale for the volumes used in a number of the studies in Table 3 is unclear given that doses were administered mostly via tubing, suggesting a targeted delivery to the upper nasal passage was desired. Rats commonly received between 100 to 200 µL of fluid in their nasal passages, with a mouse also receiving a 100 µL dose, suggesting that the nasal passages would have been saturated with formulation, and inhalation or swallowing would have been extremely likely. Others, usually purely pharmacokinetic studies utilising mice or rats, used total volumes of 14 to 27 µL, which are more in line with the above guidelines and may be more likely to detect direct olfactory delivery to the brain.

**Table 2:** Summary of studies that used pharmaceutical formulation for direct nose-to-brain delivery of ASMs. ✓ = toxicity was conducted; X = toxicity was not conducted.

ASD	Delivery System	Materials	Tox.	PK	Efficacy	Ref.
CBZ	Gel	Carbopol 974P (mucoadhesive polymer, hypromellose, pH 7.4	X	✓	X	[124]
	Thermo-reversible gel	Carbopol 974P (mucoadhesive polymer), Pluronic F127	X	✓	X	[125]
	Mucoadhesive *o*/*w* nanoemulgel	Oleic acid, Labrasol, xanthan gum (anionic mucoadhesive polymer)	X	X	✓	[125]
	Microemulsion	Oleic acid, Tween 80, Propylene glycol	✓	X	✓	[113]
		Oleoyl polyoylglycerides, Polyoxyl 40 hydrogenated castor oil, Diethylene glycol monoethyl ether, Polycarbopil (mucoadhesive)	✓	✓	X	[126]
	Polymeric nanoparticles	Carboxymethyl chitosan	X	✓	X	[127]
LMT	Thermo-reversible gel	Carbopol 974P (mucoadhesive polymer), Pluronic F127	X	✓	X	[128]
	Microemulsion	Glyceryl monostearate, Oleic acid, Tween 80, Pluronic P188	X	✓	✓	[111]
	Microspheres (as suspension)	Chitosan, glutaraldehyde	✓	X	✓	[129]
	Nanoliposomes	Phospholipon 90G, cholesterol	✓ (in vitro)	X	X	[130]
	Polymeric nanoparticles	PLGA and Poloxamer 407	✓	✓	✓	[131]
PHT	Microemulsion	Capmul MCM (glyceryl monocaprylate), Labrasol, PEG-8 caprylic/capric glycerides and Transcutol (diethylene glycol monoethyl ether)	✓	✓	✓	[112]
	Nanoparticles	Lecithin-chitosan	X	✓	✓	[132]
PBT	Gel	Carbopol 974P (mucoadhesive polymer, hypromellose, pH 9.5	X	✓	✓	[123]
LZM	Lipid nanoparticles in a gel	Glycerol monostearate, oleic acid and Tween 80, chitosan, Pluronic F127, β-glycerol phosphate disodium salt pentahydrate	X	✓	X	[133]
DZP	Polymeric nanoparticles	PLGA (Poly(D,L-lactide-co-glycolide), Pluronic F127	X	✓	X	[134]
TRH	Polymeric nanoparticles	PLA (Polylactide)	X	X	✓	[17,18]
VA	Lipid nanoparticles	Cetyl palmitate, soy lecithin, octyldodecanol	X	✓	✓	[135]
LVT	Thermo-reversible gel	Pluronic F127; Carbopol 974P and Noveon^®^ Polycarbophil.	✓	✓	X	[136]
ZNA	Thermo-reversible gel	Pluronic F127; Carbopol 974P and Noveon^®^ Polycarbophil	X	✓	X	[137]

Tox = toxicity; PK = pharmacokinetic. CBZ = carbamazepine; LMT = lamotrigine; PHT = phenytoin; PBT = phenobarbital; LZM = lorazepam; DZP = diazepam; THR = Thyrotropin releasing hormone; VA = valproic acid; LVT = levetiracetam; ZNA = zonisamide.

While light sedation is necessary to perform most intranasal administration procedures on rodents [73], it should be noted that anaesthesia (mainly long-acting anaesthesia) has been suggested to increase nasal absorption of therapeutics in rats. This is most likely due to impairment of mucociliary clearance and decreased losses due to drainage and mechanical removal (e.g., sneezing/snorting) in the conscious state [122]. For this reason, results may overestimate the true absorption that would be expected in a conscious animal. Nonetheless, such studies are still a valuable screening tool with which to assess nasal absorption of different therapeutics. Several studies in Table 3 utilised anaesthesia in the dose administration process. A number of studies did not report either way, but considering the volumes administered, almost certainly would have required it, while another claimed to have administered a gel via tubing to conscious rats. In addition to the effect on anaesthesia on mucociliary clearance, efficacy studies of intranasal anti-seizure therapeutics must also control for another potential confounder, the effect of anaesthetics on seizure threshold, which will return in our later discussion.

**Table 3:** Summary of intranasal doses and administration methods used in studies investigating intranasally-delivered ASMs.

Drug	Animal Model	Dose (µg)	Volume	Anaesthesia	Method	Ref.
CBZ	Mouse	12 to 16	12 to 16 µL in one nostril	Ketamine and xylazine (i.p.)	Tubing	[128]
		625	100 µL in one nostril	Diethyl ether	Cannula strengthened by jacketed non-protruding needle	[125]
		40 to 60	25 µL in each nostril	Not reported	Tubing	[127]
	Rat	35 to 40	10 µL in each nostril	Ketamine (i.m.)	Tubing	[126]
		50 (administered) 40 (accepted)	50 mg gel into one nostril. Estimated that 80% was accepted	None	Tubing	[124]
		1600 to 2000	55 µL in each nostril	Not reported	Tubing	[113]
LMT	Mouse	110 to 125	Not reported Both nostrils.	Ketamine and xylazine (route not stated)	Tubing	[129]
		120 to 160	12 to 16 µL in one nostril	Ketamine and xylazine (i.p.)	Tubing	[138]
	Rat	720 to 970	100 µL in each nostril	Ketamine (i.m.)	Not reported	[111]
		166 to 291	Not reported	Not reported	Not reported	[131]
PHT	Rat	3520	88 µL in each nostril	Not reported	Tubing	[112]
	Mouse	280 to 420	60 µL (number of nostrils not reported)	None	Dropper	[132]
PBT	Rat	1100 to 1200 2000 to 2200 6000 to 6600	7 to 40 µL in each nostril	Propofol (i.v.)	Deposited at opening of nares or using tubing	[123]
LZM	Rat	200	50 µL in each nostril	Not reported	Tubing	[133]
VA	Rat	720 to 840	100 µL in each nostril over a few minutes	Light ether	Tubing	[135]
DZP	Rat	40 to 50	10 µL each nostril	Ketamine (i.p.)	Tubing	[134]
TRH	Rat	20	25 µL in each nostril (chronic administration)	Isoflurane	Surgically inserted cannulae	[17,18]
LVT	Mouse	625	25 µL (left nostril only)	Ketamine and xylazine (i.p.)	MicroSprayer^®^ Aerosolizer coupled to a high-pressure syringe	[136]
ZNA	Mouse	418 to 501	50 µL (left nostril only)	Ketamine and xylazine (i.p.)	MicroSprayer^®^ Aerosolizer coupled to a high-pressure syringe	[137]

CBZ = carbamazepine; LMT = lamotrigine; PHT = phenytoin; PBT = phenobarbital; LZM = lorazepam; VA = valproic acid; DZP = diazepam; THR = Thyrotropin releasing hormone; LVT = levetiracetam; ZNA = zonisamide. i.v. = intravenous; p.o. = per oral; i.p. = intraperitoneal; i.m. = intramuscular.

#### 7.2.2. Adverse Effects and Toxicity

Intranasal drug delivery studies in general seem to give poor attention to adverse effects or toxicity of administered formulations to the nasal mucosa [139]. While this is an aspect that seems to be brushed over in preclinical trials, it is important to consider, especially in terms of exploiting a direct nose-to-brain pathway. Kozlovskaya et al., suggested that the fractions of drug reportedly delivered to the brain intranasally in a number of studies were so substantial that they implied a breach of physiological barriers by formulation constituents (e.g., permeation enhancers and co-solvents) [84]. Furthermore, they speculated about the toxicity that could potentially result from chronic exposure of olfactory or trigeminal neurons to drugs or particles transported via intracellular routes. To consider adverse effects as a whole, one must evaluate both behavioural and histological aspects. While the former receives a lot of attention in human trials [62], preclinical studies offer an excellent opportunity to screen histologically and optimise dosage and formulation, given the great similarities between rodent and human nasal epithelia [70] and the extensive guidelines on nasal tissue processing and evaluation [70,74,140,141]. Most of the studies reviewed in Table 2 did not report any data on either behavioural or histological adverse effects, and those that did presented low quality images of nasal mucosa exposed in vitro without any indication as to what type of epithelium or anatomical structures were shown [112,113,129,142]. Given that all studies performed in vivo experiments in rodents, be they pharmacokinetic, pharmacodynamic, or both, there seems no reason why the nasal passages of those rats could not have been dissected after the experiment and histologically processed to provide a substantially more meaningful evaluation of epithelial integrity.

Behavioural assessment is also important in rodents as, aside from ethical considerations and determining how much formulation an animal can feasibly tolerate in its nasal passage, it may draw attention to a highly irritant formulation and has implications for follow-up studies, such as evaluation of anti-seizure effects, given that susceptibility to these might be altered by stress or pain secondary to a nasal administration. Therefore, this appears to be an area which deserves more attention in intranasal studies.

#### 7.2.3. Quantification of Drug in Tissues

The efficiency of intranasal delivery is most adequately assessed by calculation of two parameters, (Equations (1) and (2)) [84]. The first is Drug Targeting Efficiency percentage (%DTE), which is the relative exposure of the brain to the drug following intranasal and systemic administration. The second is the nose-to-brain Direct Transport Percentage (%DTP), which is the percentage of the dose that is estimated to reach the brain via direct routes compared with the overall delivery to the brain. A %DTE greater than 100% indicates better overall brain delivery via the intranasal route compared the parenteral route, while a %DTP greater than 0% indicates an increased efficiency of brain delivery by direct routes (e.g., olfactory and trigeminal pathways) [84].

(1) $$\%DTE=\frac{(AUC_{Brain}/AUC_{Blood})in}{(AUC_{Brain}/AUC_{Blood})iv}\times 100$$

Equation (1)—calculation of Drug Targeting Efficiency (%*DTE*). *AUC_Brain_* = *AUC* (concentration versus time) for brain; *AUC_Blood_* = *AUC* (concentration versus time) for blood.

(2) $$\%DTP=\frac{(Brain_{in}/Brain_{X})in}{Brain_{in}}\times 100\;\;\;\;\;\;\;B_{X}=\frac{B_{iv}}{P_{iv}}\times P_{in}$$

Equation (2)—calculation of Direct Transport Percentage (%*DTP*). *B_in_* = brain AUC over time after intranasal administration; *B_X_* = brain AUC fraction contributed by systemic circulation through blood–brain barrier after intranasal administration; *P_in_* = blood AUC over time following intranasal administration; *P_iv_* = blood AUC over time following intravenous administration.

Calculation of these parameters is based on the following assumptions [84]. Drug pharmacokinetics are assumed to be linear, no saturation of individual absorption, distribution, metabolism, or elimination processes. *AUC_brain_* and *AUC_blood_* are assumed to reflect pharmacologically relevant drug concentrations in the brain and blood, respectively, despite (1) that drug can exist in several forms in these sites (e.g., particle-based formulations may exist as free, protein-bound or encapsulated drug) and (2) differences in intra-brain disposition of the drug, as a result of reaching the brain via different routes. The latter can be remedied by microdissection of different brain regions, and the former may possibly be addressed with analytical methods.

Kozlovskaya et al. [84] reviewed all nose-to-brain delivery studies available in February 2014 and found that only 3.1% contained the pharmacokinetic information required to calculate in vivo AUC of concentration vs. time for both brain and systemic circulation after intranasal and parenteral routes, respectively. They noted that the drug was in most cases not completely eliminated at the last sampling point (8 to 24 h), introducing error into drug exposure calculations derived from partial curves. With this is mind, we turn to the studies involving anti-seizure therapeutics that presented pharmacokinetic data, a number of which have been published since that time.

There have been some remarkable claims made about the intranasal delivery of anti-seizure therapeutics in recent years, but, unfortunately, the designs of the studies (Table 4) and the non-standardised reporting of results make the pharmacokinetic data difficult to interpret. Eskandari et al. [135] reported a brain:plasma ratio of around 8 for valproic acid delivered in intranasal lipid nanoparticles (4 mg/kg), compared to a ratio of <1 from an intraperitoneal control (150 mg/kg) at 60 min after administration. The different doses used, the single time point evaluation, and the use of intraperitoneal administration as a control impede assessment of contribution from the direct pathway that the authors claim was demonstrated in these results. Though it is likely that sustained release from the lipidic formulation and nasal absorption played a role in the differences seen, one may speculate that the control was disadvantaged at 60 min, considering that this time point equates to the lower limit of the half-life of valproic acid in rats [143] (Table 5). Acharya et al. [112] assessed an intranasally administered microemulsion containing phenytoin. The authors reported higher levels of phenytoin in the brain after the intranasal microemulsion compared to intraperitoneal phenytoin solution at 15 and 30 min following administration. Again, this study suffered from an intraperitoneal control and insufficient time points to calculate any pharmacokinetic parameters. There was also no data provided on plasma concentrations. Alam et al. [111] also assessed a type of lipid nanoparticle, this time with lamotrigine. Once again, there was no intravenous (or even intraperitoneal) control and measurements were performed at only one time point, 24 h after administration, with the intent of demonstrating a sustained effect of drug delivered with the intranasal formulation. Plasma concentration was higher than the brain concentration at this time. Some of the studies also employed intranasal solutions of their respective ASMs, which performed better than the systemic controls, suggesting that intranasal delivery of the free drug solution did occur, but that the formulations appeared to enhance drug delivery in some way, at least at the time point tested.

Some studies assessed intranasal particle delivery in more detail, however, still with setbacks. Patel et al. [126] studied an intranasal microemulsion (again, more correctly, a nanoemulsion) containing carbamazepine, both with and without a mucoadhesive agent to aid retention in the nasal passage. They studied time points from 30 to 480 min after administration and reported a carbamazepine %DTE of 241, 188, and 110 for their mucoadhesive microemulsion, microemulsion, and carbamazepine solution, respectively. Similarly, %DTP values were reported as 59, 47, and 9. However, the intravenous control values from which these values were calculated was based on intravenous administration of the microemulsion, rather than free drug solution, which likely had significant effects on the pharmacokinetic profile. Evidence of this is implied in the supporting gamma scintigraphy images, which suggests both an extravasation in the tail vein where they were injected and an extensive accumulation of the emulsion particles in the liver. Sharma et al. [134] studied the delivery of diazepam with polymeric nanoparticles with reported mucoadhesive properties, also covering a time range of 30 to 480 min. The authors reported a %DTE of 258 for the intranasal nanoparticles and 125 for the intranasal drug solution, while %DTP values were 61.3 and 1. The intranasal nanoparticles resulted in brain levels higher than intravenous and intranasal solutions from 30 min onwards. Despite this more encouraging indication of direct delivery, it should be noted that both studies derived their “drug quantification” data indirectly from scintillation measurements of Technetium-99m in tissues, thus the values do not unequivocally represent actual quantities of the drugs concerned.

The most extensive studies in this area have been reported with intranasal gels comprising the mucoadhesive polymer Carbopol 974P. Barakat et al. [124] began by testing such a gel loaded with carbamazepine over 5 to 120 min after administration. The authors reported a peak in intranasal concentrations in the brain at 5 min after administration (brain:plasma ratio of around 10), which significantly exceeded plasma concentrations for up to 20 min after administration. The intravenous control was administered at a 40 times higher dose (8 mg/kg vs. 0.2 mg/kg), but brain levels did not peak until 20 min and C_max_ was 4.5-fold lower than intranasal. Despite these intriguing results, an intravenous comparison with an equivalent dose to that administered intranasal would have been useful for direct comparison, especially considering that higher doses of carbamazepine can lead to induction of its own metabolism [147], which could have potential to alter the systemic pharmacokinetic profile. Czapp et al. [123] followed with a study of a gel containing phenobarbital, recording two types of pharmacokinetic data. The first was microdialysis in the frontal cortex extracellular space from 15 to 240 min. The gel provided a higher drug concentration in the dialysate than intranasal or intravenous control solutions, which was significantly different from 30 min onwards, but the plasma:dialysate ratio was not significantly different after this. The second method was the classic brain homogenisation from 2 to 240 min, although different regions were microdissected and analysed to provide more detailed information at 10 min. It was observed that whole brain concentrations rapidly increased during the first 10 min after gel administration, but so too did plasma concentrations. Ultimately, no difference in whole brain penetration rates between intranasal and intravenous administration was found. Upon microdissection at 10 min, however, the olfactory bulbs had a 3-fold higher concentration after intranasal gel administration. Concentrations in other brain regions, however, including those implicated in trigeminal nerve delivery routes (e.g., pons), remained similar or even decreased compared with intravenous. Despite this, it is interesting to consider their finding of respiratory centre depression at high doses of the intranasal gel that was not seen after intravenous administration, which might imply selective delivery to this area. However, the brains of these animals were not analysed, and the authors instead attributed this to increased toxic metabolites reaching the brain due to shorter systemic exposure.

More recently, Serraheiro et al. [128,138] investigated intranasal gels containing carbamazepine or lamotrigine administered to mice. The calculated %DTE in both experiments were 96% and 98%, respectively, which implied equivalent overall drug delivery to the brain by both intranasal and intravenous routes. The concentration vs. time plots reported for lamotrigine showed the intravenous administration resulted in a shorter T_max_ (5 min vs. 45 min) and greater C_max_ in the brain; however, upon microdissection into olfactory bulbs, frontal cortex, and remaining brain, the intranasal profile revealed significant heterogeneity between the regions. In line with the observations of Czapp et al. [123], markedly elevated concentrations (25 to 67 fold) in the olfactory bulbs at 5 and 10 min relative to the other brain regions was observed. Importantly, this remained elevated above plasma levels, suggesting an alternative source of penetration. Concentrations in the rest of the brain appeared to steadily increase over the time but did not reach the magnitude of that seen in the olfactory bulbs. Had other regions of the brain been dissected and analysed separately, it may have revealed further differences, although the results of Czapp et al., would suggest otherwise [123]. In the case of carbamazepine, the results were not so profound. Whilst the authors reported that higher values in the olfactory bulbs and frontal cortex up to 15 min after administration was observed, but unlike lamotrigine, these were marginally above plasma levels and very similar to concentrations seen after intravenous administration. Given that these two experiments were performed by the same lab, it would suggest different behaviour of the drug molecule, perhaps highlighting different absorption routes and brain distribution patterns. While the study of Barakat et al. [124] discussed earlier would appear to contest this, the fact that it was performed in rats may suggest an interspecies difference. Alternatively, the differences may be an indication that, from a pharmaceutical perspective, one thermo-reversible gel does not fit all, and the interactions of different therapeutics with a delivery system may significantly affect their in vivo performance.

From the above discussion, it is evident that the pharmacokinetics of intranasal anti-seizure therapeutics has a foundation, but there are clearly improvements and further discoveries to be made. Firstly, it is interesting to note that the claims of enhanced brain delivery from the particle-based studies are all based on the analysis of whole brains, rather than microdissected ones, which the gel studies asserted were required to identify significantly elevated concentrations compared to plasma. Whether this is because of the proposed benefit of particles over gels by Kozlovskaya et al. [84] or simply the methodological shortcomings of the particle studies is unclear, and further, more comprehensive, and objective studies with these systems are clearly needed to begin answering these questions. Furthermore, routine pharmacokinetic analysis of different brain regions, particularly those of relevance to olfactory and trigeminal pathways (e.g., olfactory bulbs and brainstem) will further elucidate the roles of different pathways in nose-to-brain transport and how they might be best utilised to treat neurological diseases such as epilepsy. Another factor to consider is the therapeutic relevance of the concentrations reported to reach the brain. While not detailed in this review, ASMs have been studied for many decades and a deeper literature search will reveal what is considered as a therapeutic brain concentration in a given animal model. Intranasal delivery systems that hope to be translated for human use one day for the delivery of existing ASMs should consider whether the doses delivered via direct pathways are relevant to the treatment of seizures when reporting their results. Although in the case of heterogeneous delivery, this may be difficult, so will require efficacy studies and validated positive controls, which will ultimately be necessary on the pathway to translation anyway. Finally, putting the direct-pathway-only mentality aside, one may speculate that if you can successfully exploit a direct nose-to-brain route through administration of a lower overall dose than is required systemically (i.e., achieve a %DTP above 0) and deliver sufficient therapeutic concentrations directly to key brain regions (e.g., olfactory bulbs), then perhaps it does not matter if some is absorbed systemically, provided that the systemic exposure is low enough that it will not have any significant adverse effects.

#### 7.2.4. Qualitative Distribution in Tissue

To supplement (or replace in some cases) tissue quantification data, a few of the listed studies performed gamma scintigraphy using formulations labelled with Technetium-99 m [111,112,126,134], which was reportedly associated with the drug. Acharya et al. [112] provided images of rats after intranasal phenytoin microemulsion and intraperitoneal phenytoin solution. The authors reported accumulation of intravenous phenytoin in the liver and spleen, while the intranasal microemulsion was associated with the brain and respiratory tract. Similarly, Patel et al. [126] presented images after administration of intravenous microemulsion, intranasal microemulsion, intranasal mucoadhesive microemulsion, and intranasal carbamazepine solution. The authors reported that brain distribution was higher with intranasal compared with intravenous administrations, particularly for the mucoadhesive formulation, but the image quality obscures the shape of the animal and possibly even exhibits different scales. Finally, Alam et al. [111] presented images of a rat at different time points after intranasal lipid nanoparticle administration describing an initial deposition in the nostrils that moves to the brain. They also noted a significant portion in the oesophagus and abdominal region. While this data complements the pharmacokinetic studies to an extent, it is very difficult to discern where the drug is depositing, especially given that the brain sits directly above where the liquid formulation is initially deposited. Comparisons of relative intravenous and intranasal brain distribution, therefore, was not useful.

Kubek et al. [17], on the other hand, used fluorescent illumination (exact procedure not specified) to examine the brains of animals after administration of polymeric nanoparticles containing Nile Red. This was necessary as, due to the endogenous nature of their therapeutic molecule, TRH, the exogenously administered peptide could not be directly quantified and distinguished from the endogenous peptide in the brain tissue. Based on the Nile Red fluorescence, the authors claimed widespread distribution and sustained presence of nanoparticles within the brain for up to 96 h. However, the control presented was a rat exposed to larger-sized nanoparticles at 24 h, rather than Nile Red alone. Given that free Nile Red will emit a red wavelength on contact with polar membrane lipids [148], such as the extensive array found in the brain, the claim that this represented the presence of nanoparticles is debatable. Furthermore, an immunohistochemistry assay was developed to detect the nanoparticle polymer in the brain, but only a validation of the assay after intra-amygdala injection of the nanoparticles, rather than their detection in the brain of an intranasally-treated animal was reported.

Thus, while qualitative data may provide a supplement to quantified drug distribution patterns, it was still inadequate, at least in the ways it was used in the reviewed studies, to convincingly demonstrate the existence of a direct nose to brain pathway. The gamma-scintigraphy studies did, however, provide an interesting insight into the distribution of the formulation into other body regions after the intranasal administration (e.g., possible swallowing or inhalation), indicating that the administration and volume could be optimised further. However, it should be noted that the animals were anaesthetised so that they could be imaged, which likely changed the distribution compared to when they were conscious, especially in terms of clearance from the nasal cavity, which may explain partially why the label appears to remain in the head area.

#### 7.2.5. Efficacy

Another important parameter in any drug delivery study is demonstrating pharmacodynamic efficacy in an animal model. As discussed in the previous section, a number of clinically-predictive seizure models have been designed to provide a platform for high throughput and cost effective screening of anti-seizure therapeutics. Table 2 shows that the intranasal formulation literature to date has largely focused on the recapitulation of well-characterised anti-seizure drugs. This provides an advantage as far as efficacy testing is concerned in that one can predict which seizure models will be most useful for testing whether these molecules are reaching the brain in sufficient concentrations to elicit an effect. The discussion in the previous section also highlights how one might predict, based on the theory of direct nose-to-brain pathways, which models may be most useful for detecting these specific effects from direct nasal delivery routes and, thereby, fit the model to the research question.

Of the studies summarised in Table 4 that employed a seizure model, most performed the MES test or unvalidated variations thereof or the s.c. PTZ test, whilst two used an amygdala kindling model. Aside from the kindling studies, no rationale was provided for why a specific model or seizure endpoint was chosen. In most cases, the model chosen was at least relevant to the drug being tested, but in the case of carbamazepine and lamotrigine-based formulation testing in the PTZ model, this is unclear [15]. While most reported anti-seizure effects, in a lot of cases this could not be reliably attributed to anything other than an enhancement of systemic absorption, especially in the cases where efficacy claims were not accompanied by pharmacokinetic data [113,125,129]. Furthermore, considering the lack of adverse effect and toxicity data provided, as discussed above, it is possible that damage to physiological barriers could have been a major contributor to any enhanced delivery. Nonetheless, like the pharmacokinetics data, and barring the limitations, the studies would seem to support the use of the nose as a rapid and sustained method of drug delivery.

The end points used were standard in most studies, except in the case of MES variations, which were not justified. Samia et al. [125] reported data based on protocols that employed extremely lengthy stimulations and used stimulations until death as an endpoint, which has no scientific basis. Eskandari et al. [135] used parameters of 110 mA, 100 Hz, 1 ms pulse width, and 0.2 s shock duration, validating their method with a very high dose of intraperitoneal phenytoin (90 mg/kg), on the basis that more than 50% of rats displayed extension when untreated, but none did when treated. Given that less than 100% of untreated rats displayed extension, it is unclear, firstly, how a decrease in extension:flexion ratio compared to control as an endpoint was used; and, secondly, why the authors did not simply report it as flexion:extension ratio; and, furthermore, why the decrease in this parameter appeared to be almost as high as the drug-treated rats in those given blank nanoparticles. Although not commented on by the authors, this might suggest that the components of the formulation itself may have played a role in eliciting the apparent anti-seizure effects, as has been reported elsewhere [118]. Alternatively, it is also possible this may have been related to the anaesthesia (reported as ‘light ether’) used during administration that the untreated controls may not have received.

In fact, most studies who reported using anaesthesia did not comment on whether this was also applied to untreated controls to which efficacy data were compared or normalised to account for a potential confounding effect on seizure threshold [111,125,129,135]. For short-acting inhaled anaesthesia, this was likely less influential, but certainly with systemically-administered longer-acting anaesthetics, ketamine [149,150], and propofol [151], this may have been an important confounder in tests performed 60 min or less after administration. Czapp et al. [123], however, specifically stated that the administration of propofol alone in preliminary experiments did not affect kindling parameters and also administered anaesthesia to all intravenous controls. They did, however, require increased doses of propofol to administer increased doses of nasal gel (containing more phenobarbital), and considering the synergistic interaction previously reported between propofol and phenobarbital [152], it should be noted that this may have contributed somewhat to the significant anti-seizure effects noted at the higher, but not the lower, dose after intranasal administration.

The most used time point for testing, regardless of the ASM studied, was 60 min. Given that a key aim of intranasal delivery is to exploit direct and rapid routes to the brain, which the pharmacokinetic data discussed in the previous section provide evidence for, the rationale for the popularity of this time point was unclear. In some cases, such as that of Czapp et al. [123], where kindling parameters were measured 60 min after administration, it may have been related to the fact that substantial anaesthesia was used during the administration (in that case, intravenous propofol), necessitating a significant time delay to allow the animals to regain consciousness before stimulations. Alternatively, it may have been based on the time to peak effect of systemic phenobarbital [91], but given that relatively high concentrations were found in the olfactory bulbs at 10 min after intranasal compared with intravenous administration, an earlier time point would have been interesting if it were possible. Only one study performed stimulations at a range of time points (15 to 120 min) to determine a time of peak effect [135], which would have been useful in other studies given that nasal administration may change the pharmacokinetics of an ASM. The parenteral half-lives and times to peak effect of the ASMs used in the reviewed studies are included for the reader’s reference in Table 5.

#### 7.2.6. In the Pipeline

Outside of the published literature, pharmaceutical formulation for the intranasal treatment of seizures is also gaining traction, particularly in the arena of cannabinoids [153]. The potential role of these molecules in the treatment of pharmacoresistant epilepsy [154] is an area of much current interest, but their inherent lipophilicity and low bioavailability makes delivery an issue [155]. Intranasal pharmaceuticals are being explored to address this challenge, albeit not specifically for seizures, but neurological conditions in general [156]. While this area is still young, formulation is increasingly being recognised as a requirement to exploit intranasal delivery, and innovative therapeutics, to their fullest.

## 8. Conclusions

This review highlights some important considerations that should be addressed to further the exploration of the field of intranasal treatment of seizures with the development of pharmaceutical formulations. Gels would seem to be the most well-characterised pharmacokinetically for the delivery of ASMs, but a review of the wider intranasal formulation literature suggests that particulate delivery systems may be an important player once they are more rigorously studied. With all its shortcomings, the existing literature would suggest that there are advantages to delivering ASMs through the nose, but which direct pathways (if any) are able to be exploited and whether this can be achieved without damaging the nasal mucosa, as well as whether it is a feasible chronic treatment, is yet to be determined. More attention to obtaining quality and hypothesis-driven pharmacokinetic and pharmacodynamic data with suitable controls, as well as more detailed and standardised reporting of methodology, should contribute a lot towards answering these questions.

## Figures and Tables

**Table 1:** Comparison between key aspects of the rat and human nasal passages. Based on Kapoor et al. [22], Lochhead & Thorne [36] and Illum [75,83] with reference to a 70 kg human and a 250 g rat. CSF = Cerebrospinal fluid.

Parameter	Human	Rat
Nasal cavity volume	25 cm^3^	0.26 to 0.4 cm^3^
Nasal cavity surface area	150 to160 cm^2^	13.4 to 14 cm^2^
Surface area per unit volume	6.4	51.5
Olfactory epithelium area (area, %)	12.5 cm^2^, 8%	6.75 cm^2^, 50%
CSF volume	160 mL	150 µL
CSF volume replacement frequency	5 h	Hourly
Shape of upper airways	L-shaped	Linear
Type of breathing at rest	Oronasal	Obligate nose
Connection between nasal cavity and oral cavity	No (incisive canal is not patent)	Yes (nasopalatine canal is patent)
Vascular swell bodies in septum	No	Yes
Turbinates (number and shape)	3; comma shaped	3; t-shaped with elaborate scrolls
Presence of ethmoid sinuses (air cells) and spheroid sinuses	Yes	No
Maxillary sinuses	Large; open	Small; closed
Nasal secretion movement	Mostly posteriorly (to nasopharynx)	Mostly anteriorly (towards nostril)
Inspiratory airflow route	Close to floor of nasal passage	Upward and laterally

**Table 4:** Summary of methodology used in studies analysing pharmacokinetics and anti-seizure efficacy of intranasally-delivered ASMs.

ASD	Tissues Analysed	PK Parameters Reported	Time Points after Administration	Routes/Formulations Compared	Test	Endpoint	Time of Test	Anaesthesia	Ref.
CBZ	Brain—olfactory bulbs, frontal cortex, remainder; plasma; liver	DTE; brain, plasma and liver concentration; B:P; T_max_; C_max_; AUC; k_el_; t_1/2_, MRT; F	5, 10, 15, 30, 60 min	i.n. (form.); i.v. (form.)	-	-	-	Ketamine and xylazine (i.p.)	[128]
	Brain; plasma	Brain and plasma concentration; AUC; T_max_; C_max_; k_el_; t_1/2_; %DTE; %DTP	30, 60, 120, 240, 480 min	i.n. (form. x 2); i.n. (sol.); i.v. (form.)		-	-	Ketamine (i.m.)	[126]
	Brain; plasma	Brain and plasma concentration; B:P ratio; AUC; C_max_; T_max_; MRT, AUC (B:P)	5, 10, 15, 20, 30, 45, 60, 90, 120 min	No treatment; i.n. (sol.); i.n. (form.); p.o. (sol.)	-	-	-	None	[124]
	-	-	-	i.n. (sol.); i.n. (form.); p.o. (form.); i.n. (sol.); No treatment	MES (auricular)	Duration of HLE	60 min	None reported	[113]
	-	-	-	i.n. (form.); i.n. (sol.); No treatment	MES variant (auricular) PTZ (i.p.)	MES variant: number of trials until death PTZ: onset to convulsion, time until death	5 min (MES variant) 15 min (PTZ)	Diethyl ether	[125]
	Brain; plasma	Brain and plasma concentration; AUC; T_max_; C_max_; MRT	0.25, 0.5, 1, 2, 3, 4 h	i.n. (form); i.n. (sol.)	-	-	-	None	[127]
DZP	Brain, plasma	%DTE; brain and plasma concentration; C_max_; T_max_; AUC	30, 60, 120, 240, 480 min	i.n. (sol.); i.n. (form.); i.v. (sol.)	-	-	-	Ketamine (i.p.)	[134]
VA	Brain; plasma	Brain and plasma concentration; B:P	60 min	i.n. (form. no drug); i.n. (form.); i.n. (sol.); i.p. (form. no drug); i.p. (form.); i.p. (sol.)	MES variation (auricular)	E:F ratio of hindlimbs	15, 30, 60, 90, 120 min	Light ether	[135]
PHT	Brain	Brain concentration	15 and 30 min	No treatment; i.n. (form.); p.o. (form.); i.p. (sol.)	MES (auricular)	Duration of HLE	60 min	None reported	[112]
	Brain, serum, liver, spleen and kidneys	DTE% and DTP%; Brain, plasma and liver concentration; C_max_; T_max_; AUC_brain_/AUC_plasma_ ratio; t_1/2_	5, 15, 60, 240, 1440, 2880, 4320, 5760, 7200 min	i.n. (form); i.p. (sol)	PTZ (s.c.)	Duration; frequency; total number of EEG signal	1, 4, 48 h	Ketamine and xylazine (i.p.)	[132]
PBT	Whole brain. OB, frontal cortex, piriform cortex, amygdala, hippocampus, parahippocampal cortex, caudal cortex, cerebellum, pons. Frontal cortex dialysate; plasma.	D:P; (microdialysis in frontal cortex); brain and plasma concentration (homogenate); B:P (homogenate)	10 min (microdissected regions) 2, 5, 10, 20, 30, 60, 200, 240 min (whole brain and plasma) 15, 30, 60, 90, 120, 180, 240 min (dialysate)	i.n. (form. no drug); i.n. (form.); i.v. (form. no drug); i.v. (form.)	Amygdala kindling	ADT; seizure severity and duration; ADD; GST	60 min	Propofol (i.v.)	[123]
LMT	-	-	-	Saline (route not reported); i.n. (form.); i.p. (form.)	PTZ (s.c.)	Onset to clonic convulsion Protection against mortality	30 min	Ketamine and xylazine (route not reported)	[129]
	Brain—olfactory bulbs, frontal cortex, remainder; plasma; liver	DTE; Brain, plasma and liver concentration; B:P; C_max_; T_max_; AUC; k_el_; k; t_1/2;_ MRT; Absolute i.n. F; AUC ratio (L:P)	5, 10, 15, 30, 60, 120, 240 min	i.n. (form.); i.v. (form.)	-	-	-	Ketamine and xylazine (i.p.)	[138]
	Brain; plasma	Brain and plasma concentration	24 h	i.n. (sol.); i.n. (form.); p.o.	MES (auricular)	HLE incidence; Latency to HLE; Duration of HLE	60 min 24 h	Ketamine (i.m.)	[111]
	Brain; plasma	Brain, plasma concentration; B:P; T_max_; C_max_; AUC; i.n. F; t_1/2_, MRT; F	15, 30, 60, 120, 240, 480 min	i.n. (form.); i.n. (sol.); i.v. (sol)	PTZ (route of administration not reported)	Onset to seizure	15, 30 and 60 min	Not reported	[131]
THR	-	-	-	i.n. (form.); i.n. (form. no drug)	Amygdala kindling	ADD Number of seizures until first Stage 5; Number of seizures until fully kindled	Daily stimulations until fully kindled; Doses administered at both 60 and 30 min before stimulation	Isoflurane	[17,18]
LZM	-	-	-	i.n. (form.); i.n. (form. no drug); i.p. (sol.)	PTZ (s.c.)	Lag time of incidence; severity of symptoms in trunk (0–3); severity of symptoms in hands and feet (0–3); duration of symptoms	-	Not reported	[13]
LVT	Brain, plasm, lung and kidney	t_max_; C_max_; AUC_t_; AUC_inf_; AUC_extrap_; k_el_; t_1/2el_; MRT; F; AUC_t_; AUC_brain_/AUC_plasma_; AUC_lung_/AUC_plasma_; AUC_kidney_/AUC_plasma_	5, 15, 30, 60, 90, 120 and 240 min	i.n. (form.); i.v. (sol)				Ketamine and xylazine (i.p.)	[136]
ZNA	Brain, plasm, lung and kidney	t_max_; C_max_; AUC_t_; AUC_inf_; AUC_extrap_; k_el_; t_1/2el_; MRT; F; AUC_t_; AUC_brain_/AUC_plasma_; AUC_lung_/AUC_plasma_; AUC_kidney_/AUC_plasma_	5, 15, 30, 60, 90, 120, 240, 360, 480 and 720 min	i.n. (form.); i.n. (form. no drug); i.v. (sol); p.o. (sol.)	-	-		Ketamine and xylazine (i.p.)	[137]

CBZ = carbamazepine; DZP = diazepam; VA = valproic acid; PHT = phenytoin; PBT = phenobarbital; LMT = lamotrigine; THR = Thyrotropin releasing hormone; LZM = lorazepam; LVT = levetiracetam; ZNA = zonisamide. DTP = drug transport percentage; DTE = drug targeting efficiency; B:P = Brain:Plasma ratio; L:P = Liver:Plasma ratio; D:P = Dialysate:Plasma ratio; k_el_ = terminal elimination rate constant); k = tissue elimination rate constant; MRT = mean residence time; t_1/2_ = half-life; F = bioavailability; AUC = area under curve; AUC_extrap_ = extrapolated area under drug concentration-time curve; AUC_inf_, = area under drug concentration-time curve from time zero to infinity; AUC_t_, = Area under the concentration time-curve from time zero to the last quantifiable drug concentration; C_max_ = Maximum peak concentration; t_max_ = time to achieve the maximum peak concentration. sol. = drug in solution; form. = drug in formulation. i.n. = intranasal; i.v. = intravenous; p.o. = per oral; i.p. = intraperitoneal; i.m. = intramuscular.

**Table 5:** Half-lives and times to peak effect of ASMs used in some of the reviewed formulation studies.

ASM	Plasma Half-Life (h) [138,143,144,145]			Time (min) to Peak Effect after Single Parenteral Dose [92,146]	
	Rats	Mice	Human	Rats	Mice
Carbamazepine	1.2–3.5	30–60	25–50	30	15
Phenobarbital	9–20	7.5	70–100	60	30
Lamotrigine	12–30	8 *	21–50	60	120
Phenytoin	1–8	16	15–20	30	120
Valproic acid	1–5	0.8	8–15	15	5
Diazepam	1.4	7.7	24–72	15	15

* Estimated from plasma concentration graph in Serralheiro et al. [139].

## Data Availability

All data supporting the reported conclusions can be found in the manuscript.
